# Therapeutic Potential of Quadrigemine I Against Lymphoma: Mechanistic Insights from Cell Lines and Xenograft Models Demonstrating DNA Damage, Oxidative Stress, and Pathway Regulation

**DOI:** 10.3390/ijms26104848

**Published:** 2025-05-19

**Authors:** Junmei Pu, Wenfeng Shi, Jiabao Cui, Hui Yang, Jianxin Cao, Yaping Liu, Shanshan Xiao, Guiguang Cheng

**Affiliations:** 1 Faculty of Food Science and Engineering, Kunming University of Science and Technology, Kunming 650500, China; 20222125014@stu.kust.edu.cn (J.P.); swf821130135@163.com (W.S.); 15045227277@163.com (J.C.); 13595862451@163.com (H.Y.); 13313130@kust.edu.cn (J.C.); liuyaping@kust.edu.cn (Y.L.); 2 Yunnan Key Laboratory of Plateau Food Advanced Manufacturing, Kunming 650500, China; 3 Yunnan International Joint Laboratory of Green Food Processing, Kunming 650500, China

**Keywords:** lymphoma, quadrigemine I, xenograft mice, RNA-seq analysis

## Abstract

Lymphoma is one of the malignant tumors that significantly threatens human health. Quadrigemine I, an indole alkaloid derived from the leaves of *Psychotria pilifera*, has been studied for its potential anti-tumor properties, but its mechanisms remain poorly understood. The CCK-8 assay was used to determine the cytotoxic effect of quadrigemine I on lymphoma cells. Flow cytometry was employed to analyze apoptosis and reactive oxygen species (ROS) levels in these cells. DNA damage was assessed by the comet assay, and the underlying mechanisms were investigated using RNA sequencing (RNA-seq) and real-time quantitative PCR (RT-qPCR). The anti-tumor activity of quadrigemine I was evaluated in tumor xenograft mice. Quadrigemine I suppressed lymphoma cell proliferation with an IC_50_ of 0.46 µM. It induced apoptosis, promoted ROS generation, and caused DNA damage in tumor cells. RNA-seq analysis revealed that the significantly differentially expressed genes were notably enriched in the ErbB, p53, and apoptosis signaling pathways. RT-qPCR demonstrated altered expression levels of key genes in the aforementioned pathways. In vivo, quadrigemine I significantly inhibited tumor growth in xenograft mice by increasing apoptosis in tumor tissues, with reduced Ki-67 and Bcl-2 expression and elevated cleaved caspase-3 levels. Quadrigemine I may serve as a novel anti-tumor agent for lymphoma therapy.

## 1. Introduction

Lymphoma is a malignant tumor originating in the lymphatic system, characterized by the abnormal proliferation and spread of lymphocytes, leading to swollen lymphatic tissue and impaired organ function [1]. Clinical symptoms include enlarged lymph nodes, systemic manifestations (e.g., fever, night sweats, malaise), anemia, and thrombocytopenia [2]. The prevalence of malignant lymphoma continues to rise, making it one of the major tumor types that pose a serious threat to human health [3]. Lymphoma exhibits significant individual variability, specific symptoms, and poor treatment prognosis, with a high likelihood of recurrence within 2–3 years [4]. Therefore, recurrence and drug resistance remain major challenges in current clinical treatment, seriously threatening patients’ lives and health [5].

Common therapeutic drugs and strategies for lymphoma include chemotherapeutic agents (e.g., the CHOP regimen, ABVD regimen), targeted therapies (e.g., rituximab, belantamab mafodotin), immunotherapy (e.g., PD-1 inhibitors), and stem cell transplantation. Although these treatments are highly effective in many cases, they have certain drawbacks and limitations, such as high toxicity, susceptibility to drug resistance, high treatment costs, and long-term immune system impairment. Therefore, there is an urgent need to develop safe and effective therapeutic agents for the prevention and treatment of lymphoma [6].

Indole alkaloids are important medicinal compounds for drug exploration. To date, many indole alkaloids from plants, animals, and fungi have been developed as successful agents for clinical treatment of cancer, pain, diabetes, cardiovascular disease, and inflammatory diseases [7]. For lymphomas, vincristine, vinblastine, and camptothecin are important anti-lymphoma agents and are usually consumed with other chemotherapeutic agents [8]. Thus, indole alkaloids are important sources of new anti-lymphoma substances.

Quadrigemine I is an indole alkaloid isolated from *Psychotria pilifera*, a plant belonging to the *Rubiaceae* family. A previous study demonstrated that quadrigemine I has significant cytotoxic activity against various human cancer cells [9]. The IC_50_ of quadrigemine I was 0.56 µM for human acute promyelocytic leukemia cells (HL-60). While its effects in leukemia have been explored, its potential role in lymphoma therapy remains underinvestigated. Recent studies have suggested that indole alkaloids, such as quadrigemine I, can modulate various signaling pathways involved in cancer cell proliferation, apoptosis, and metastasis. However, the therapeutic effect of quadrigemine I on lymphomas has not yet been studied, and its possible mechanisms also need to be explored.

In this paper, the extraction, isolation, and identification of quadrigemine I were performed. The inhibitory effect of quadrigemine I on cell proliferation was evaluated in human large-cell immunoblastic lymphoma cells (SR) and Burkitt lymphoma cells (Raji) using the CCK-8 assay. In addition, the protective effect of quadrigemine I against lymphoma was assessed in an SR cell-induced lymphoma model in NOD/SCID mice. The effects of quadrigemine I on cell apoptosis, reactive oxygen species (ROS) generation, and DNA damage were systematically analyzed. Furthermore, RNA-seq analysis was conducted to identify key target genes and signaling pathways.

## 2. Results

### 2.1. Cytotoxic Effect of Indole Alkaloids on Human Lymphoma Cells and Normal Cells

**Figure 1:**
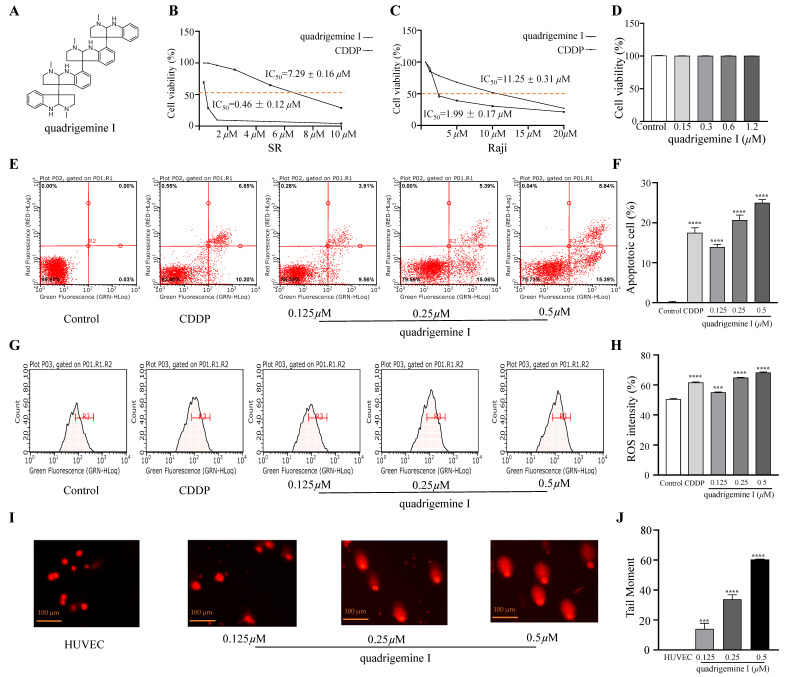
Effects of quadrigemine I on cell viability, apoptosis, ROS production, and DNA damage in SR cells. (**A**) The chemical structure of quadrigemine I. (**B**–**D**) SR cells (**B**), Raji cells (**C**), and HUVECs (**D**) were treated with different concentrations of quadrigemine I. Cell viability was evaluated by the CCK-8 assay. (**E**,**F**) Apoptosis on SR cells induced by quadrigemine I. (**G**,**H**) Intracellular ROS production in SR cells induced by quadrigemine I. (**I**,**J**) DNA comet images (scale bar: 100 μm) of SR cells induced by quadrigemine I. Data represent means ± SD from three independent experiments. *** *p* < 0.001 and **** *p* < 0.0001 compared with the control group.

We evaluated the effect of indole alkaloids on the viability of SR and Raji cells using the CCK-8 assay. Quadrigemine I exhibited concentration-dependent anti-proliferative activity in both SR and Raji cells. IC_50_ values of quadrigemine I on SR and Raji cells were 0.46 ± 0.12 μM and 1.99 ± 0.17 μM, respectively, while the positive control (CDDP) on SR and Raji cells was 7.29 ± 0.16 μM and 11.25 ± 0.31 μM, respectively (Figure 1B,C). We observed that the SR and Raji cell lines exhibited distinct responses to quadrigemine I. This difference may be attributed to their origins from different types of lymphomas—T cell and B cell, respectively. T cells and B cells possess distinct characteristics in terms of signal transduction, proliferation patterns, and apoptotic pathways, which may contribute to their differential sensitivity to quadrigemine I.

Furthermore, the effect of quadrigemine I on normal cells was evaluated by the CCK-8 assay, which showed that quadrigemine I had no significant effect on the cell viability of HUVECs at 0.5 μM, 0.25 μM, and 0.125 μM. Collectively, these results demonstrate that quadrigemine I exerts significant anti-proliferative effects on SR cells at concentrations below the cytotoxic threshold (Figure 1D).

### 2.2. Effects of Quadrigemine I on SR Cell Apoptosis

To determine the percentage of mid- and late-stage apoptotic cells, flow cytometry was performed using Annexin V-FITC/PI staining. The results showed a significant increase in mid- and late-stage apoptotic cells after quadrigemine I treatment compared to the control group (Figure 1E). Treatment with quadrigemine I for 48 h dose dependently induced cell apoptosis, as evidenced by the increase in the proportion of Annexin V-positive cells from 3.90 ± 0.01% (control group) to 14.38 ± 0.07% (1.25 μM), 21.79 ± 0.26% (2.5 μM), and 30.89 ± 1.57% (5 μM) (Figure 1F). These findings suggest that quadrigemine I reduces cell viability through inducing cell apoptosis.

### 2.3. Generation of Intracellular ROS Induced by Quadrigemine I

Excessive generation of ROS is known to trigger cellular apoptosis and/or autophagy. Therefore, we analyzed the effect of quadrigemine I on intracellular ROS production in SR cells. After treatment with quadrigemine I at various concentrations (0.125, 0.25, and 0.5 μM), a significant increase in intracellular ROS levels was observed in SR cells (Figure 1G). Quantitative analysis showed that quadrigemine I at 0, 0.125, 0.25, and 0.5 μM significantly elevated ROS levels from approximately 50.42 ± 0.45% to 55.13 ± 0.18%, 64.85 ± 0.17%, and 68.31 ± 0.31%, respectively (Figure 1H). Compared with the control group, CDDP treatment elevated ROS levels from 50.42 ± 0.45% to 61.65 ± 0.39%. These results suggest that quadrigemine I (0.25 and 0.5 μM) may trigger apoptosis through increasing ROS levels in SR cells (*p* < 0.05).

### 2.4. DNA Damage Induced by Quadrigemine I

ROS can also induce DNA damage [10]. Therefore, we further investigated the role of quadrigemine I in DNA damage in SR cells. The comet assay demonstrated that the degree of DNA damage in SR cells treated with quadrigemine I was dose dependent compared to the control group (Figure 1I). Higher concentrations of quadrigemine I led to more pronounced DNA damage in SR cells, with tail DNA percentages of 14.03 ± 0.29% (1.25 μM), 33.78 ± 0.44% (0.25 μM), and 60.39 ± 0.22% (0.5 μM) (Figure 1J). The migrated cell nuclei with DNA strand breaks exhibited teardrop, pear-shaped, or large fan-like tails. In addition, treatment of HUVECs with the same concentrations of quadrigemine I did not cause DNA damage. These findings further suggest that quadrigemine I reduced cell viability through apoptotic mechanisms.

### 2.5. RNA-Seq Analysis of Quadrigemine I-Regulated Gene Expression in SR Cells

**Figure 2:**
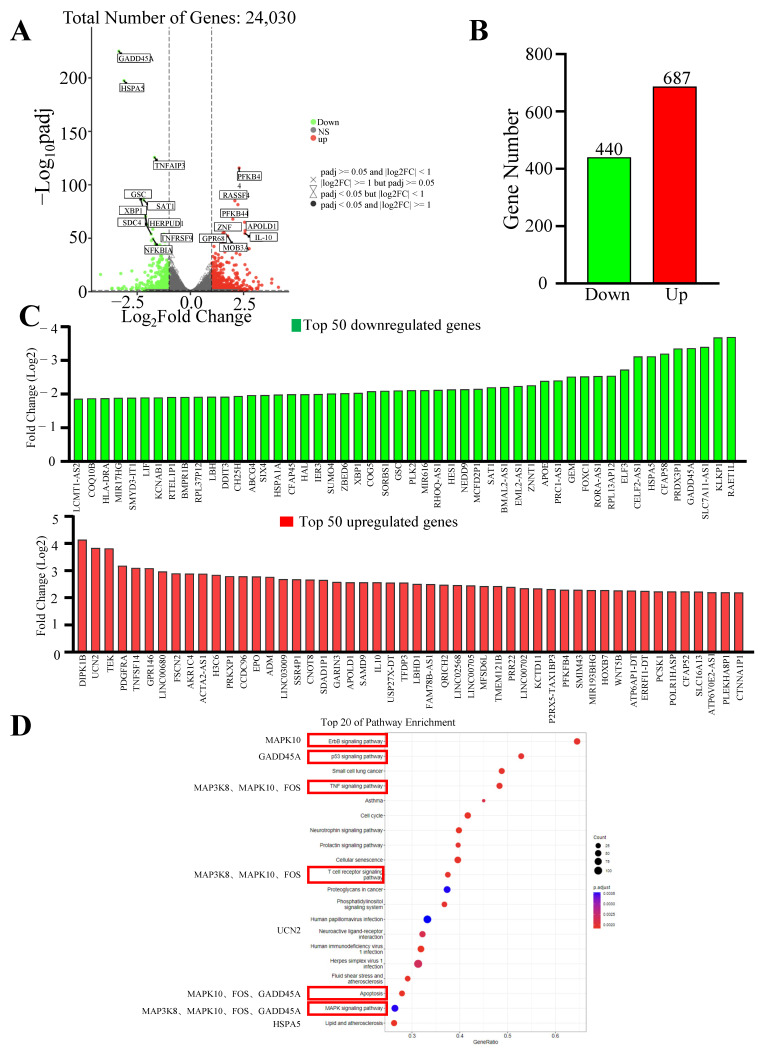
Transcriptional profiles of quadrigemine I-treated SR cells. (**A**) Volcano plots showed differentially expressed mRNAs. Green dots indicated downregulated mRNAs, red dots represented upregulated mRNAs, and gray dots showed mRNAs with nonsignificant differences. (**B**) The number of genes upregulated and downregulated by quadrigemine I. (**C**) Bar graphs showing the 18 DEGs that were most noticeably upregulated genes and downregulated genes in quadrigemine I-treated SR cells. (**D**) KEGG pathway enrichment (top 20).

To further explore the mechanism of quadrigemine I-induced apoptosis, total RNA was extracted from SR cells treated with quadrigemine I for 48 h and used for RNA-seq analysis. We compared the gene expression profiles of the quadrigemine I-treated group with those of the control group. The results exhibited that SR cells treated with 0.5 μM quadrigemine I showed substantial changes in transcriptional profiles. Therefore, we selected the high-dose group for further analysis. RNA-seq analysis showed that a total of 1127 significantly differentially expressed genes (DEGs) were identified in quadrigemine I-treated SR cells, including 687 upregulated and 440 downregulated genes (Figure 2A–C). KEGG enrichment analysis of the significantly differentially expressed genes identified a total of 129 pathways. Notably, several pathways relevant to the focus of this study were identified, including the ErbB signaling pathway, p53 signaling pathway, TNF signaling pathway, T cell receptor (TCR) signaling pathway, mitogen-activated protein kinase (MAPK) signaling pathway, and apoptosis (Figure 2D).

**Figure 3:**
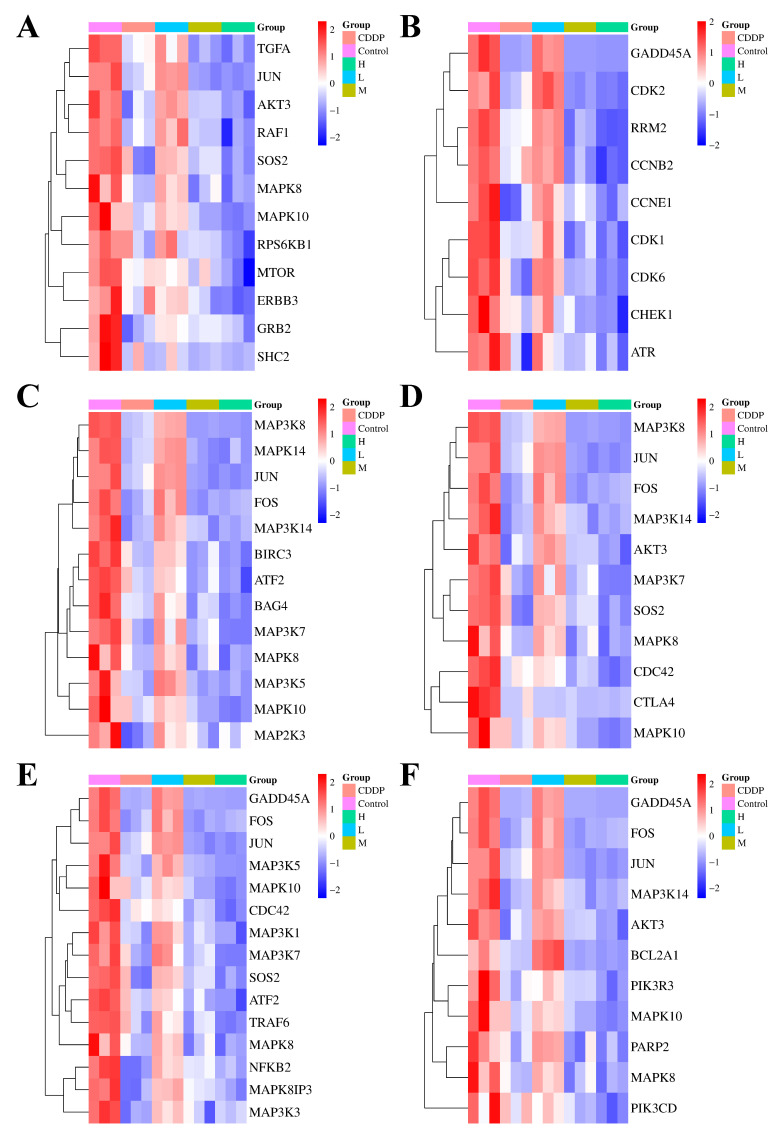
Quadrigemine I regulates the expression of genes in SR cells. (**A**) The heatmap of genes involved in the ErbB signaling pathway. (**B**) The heatmap of genes involved in the P53 signaling pathway. (**C**) The heatmap of genes involved in the TNF signaling pathway. (**D**) The heatmap of genes involved in the T cell receptor signaling pathway. (**E**) The heatmap of genes involved in the MAPK signaling pathway. (**F**) The heatmap of genes involved in the apoptosis signaling pathway. The heatmap showed all the differentially expressed mRNAs between quadrigemine I-treated SR cells (n = 3) and normal SR cells (n = 3) with adjusted *p* < 0.05 and |Log_2_FC| ≥ 1.0. Upregulated genes were presented as red dots, whereas downregulated ones were in blue.

To elucidate the specific mechanisms of action of quadrigemine I on the six candidate signaling pathways, we performed a differential analysis of the expression levels of key genes enriched in these pathways. In the ErbB signaling pathway (Figure 3A), which plays a key role in regulating cell proliferation and differentiation in various tissues and is crucial for maintaining tissue homeostasis, the ErbB family receptors are often aberrantly expressed or deregulated in tumors and play pivotal roles in cancer onset and metastatic progression. The results showed that the Erb-B2 receptor tyrosine kinase 3 (ERBB3) and transforming growth factor alpha (TGFA) genes were downregulated, suggesting that quadrigemine I treatment may block the activation of the ErbB signaling pathway, inhibit the growth and proliferation of lymphoma cells, and induce their apoptosis.

The p53 signaling pathway (Figure 3B) maintains genetic stability by participating in DNA repair, chromosome recombination, and nucleotide excision in sister chromatids, and its downregulation may affect the expression of other DNA damage repair-related genes. In our study, the growth arrest and DNA damage inducible alpha (GADD45A) gene expression level was downregulated, leading to a decline in the overall DNA repair capacity of cells.

In the TNF signaling pathway (Figure 3C), which is a crucial intracellular signaling pathway involved in immune regulation, inflammatory responses, cell proliferation, differentiation, and apoptosis, the expression of FOS and JUN genes was significantly downregulated, which may result in decreased expression of genes associated with cell cycle progression, thereby slowing the proliferation of tumor cells.

In the TCR signaling pathway (Figure 3D), which plays a central and indispensable role in the adaptive immune response by enabling T cells to recognize specific antigens and initiate appropriate immune reactions, the downregulation of AKT serine/threonine kinase 3 (AKT3) and phosphatidylinositol-4,5-bisphosphate 3-kinase catalytic subunit delta (PIK3CD) gene expression impaired the transmission of TCR signals. This disrupted the balance between normal T cell proliferation and apoptosis.

In the MAPK signaling pathway (Figure 3E), 105 genes were upregulated and 141 genes were downregulated. This pathway is a crucial intracellular signaling transduction pathway that plays a vital role in various cellular physiological processes. The MAPK subfamilies, JNK and p38 MAPK, play crucial roles in cellular apoptosis. In this pathway, genes associated with these subfamilies, such as JUN and activating transcription factor 2 (ATF2), were significantly downregulated. This may affect the expression of anti-apoptotic genes, making SR cells more susceptible to apoptosis after quadrigemine I treatment.

In the apoptosis signaling pathway (Figure 3F), the downregulation of apoptosis-related genes, such as BCL2-related protein A1 (BCL2A1), may inhibit the survival and proliferation of tumor cells, thereby suppressing tumor growth.

RNA-seq analysis revealed that seven genes related to apoptosis were altered after treating with quadrigemine I. The expression of urocortin 2 (UCN2) was evidently upregulated, with a log_2_FoldChange of 3.83, and the expression of heat shock protein family A (Hsp70) member 5 (HSPA5), MAPK10, FOS, GADD45A, E74 Like ETS transcription factor 3 (ELF3), and mitogen-activated protein kinase kinase kinase 8 (MAP3K8) was evidently downregulated in quadrigemine I-treated SR cells. The transcriptome results showed that the treatment in the high-concentration group had the best effect and could effectively reverse the expression levels of these genes, which was consistent with the previous analysis results.

**Figure 4:**
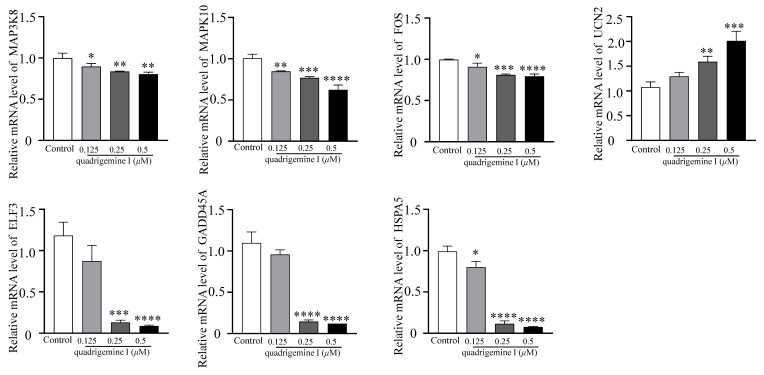
Quadrigemine I regulates the expression of genes in SR cells. Bar plots showing the expression level of the selected DEGs using RT-qPCR. n = 3; * *p* < 0.05, ** *p* < 0.01 *** *p* < 0.001, **** *p* < 0.0001.

To validate these findings, the key genes involved in the aforementioned pathways—including MAP3K8, MAPK10, FOS, UCN2, ELF3, GADD45A, and HSPA5—were selected for RT-qPCR analysis. The RT-qPCR results showed that UCN2 expression was upregulated in a dose-dependent manner following quadrigemine I treatment. In contrast, the expression levels of GADD45A, MAP3K8, MAPK10, FOS, HSPA5, and ELF3 were downregulated in a dose-dependent manner. These results were consistent with the transcriptomic data (Figure 4).

### 2.6. Safety Evaluation of Quadrigemine I in Mice

Cell experiments demonstrated that quadrigemine I inhibited the growth of SR cells in vitro. To evaluate whether quadrigemine I is a potential agent for lymphoma treatment, we first assessed its safety in vivo. Figure S1 shows the body weight and organ-to-body weight ratio of mice following continuous intraperitoneal administration of 5 mg/kg quadrigemine I over a 14-day period. All mice survived, gained weight, and displayed normal activity and health. No noticeable abnormal behaviors, adverse toxic effects, or significant changes (*p* > 0.05) in organ weights (including heart, liver, spleen, lung, kidney, and brain) were observed. The hematological analysis results of mice after intraperitoneal injection of quadrigemine I for 14 days are shown in Table S1. The serum biochemical parameters are presented in Table S2. Both hematological and biochemical indices showed no significant differences between the groups. These results suggested that quadrigemine I had no effect on liver and kidney function in mice. H&E staining revealed no pathological changes in the tissue samples (Figure S2). Therefore, it can be concluded that quadrigemine I (5 mg/kg) is safe for intraperitoneal injection in mice and is suitable for subsequent experiments.

### 2.7. Quadrigemine I Inhibits Tumor Growth in Tumor Xenograft Mice

**Figure 5:**
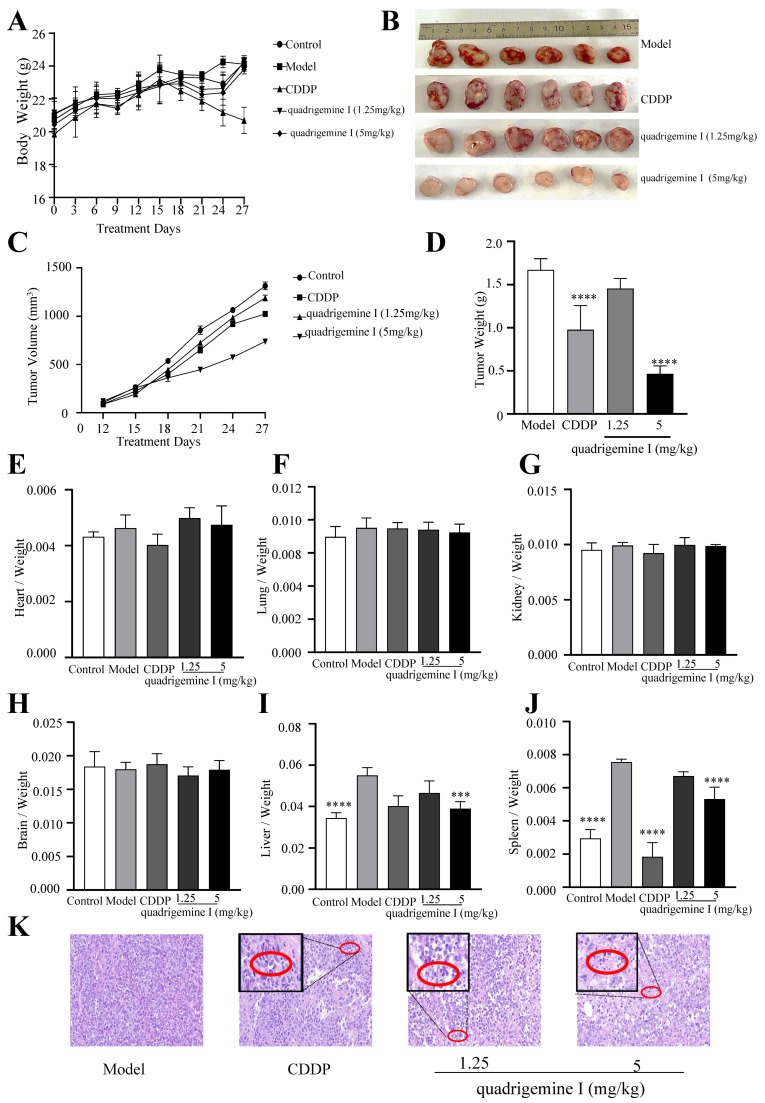
Effects of quadrigemine I on tumor growth in xenograft mice. NOD/SCID mice were subcutaneously injected with SR cells and had an intraperitoneal injection with quadrigemine I. (**A**) Body weights measured every 3 days. (**B**) Images of excised tumors. (**C**) Tumor volume. (**D**) Tumor weights. (**E**–**J**) The ratio of organs to body weight. (**K**) H&E staining of tumor tissues; the scale bar stands for 20 μm. The red circle marks apoptotic cells characterized by nuclear condensation and fragmentation. *** *p* < 0.001 and **** *p* < 0.0001 compared with the control group.

We conducted tumor xenograft assays in NOD/SCID mice to investigate the in vivo anti-lymphoma effects of quadrigemine I. Compared with the control group, the body weights of the mice did not show any significant differences throughout the experiment, except for the CDDP group (Figure 5A). Tumor growth was significantly suppressed in the quadrigemine I (5 mg/kg) group compared to the model group (Figure 5B–D). At the end of the experiment, we analyzed the organ-to-body weight ratio and found no significant differences in the heart, lung, kidney, or brain (Figure 5E–H). Notably, the organ indices of the liver and spleen in the model group were significantly increased, while treatment with quadrigemine I significantly reduced the organ indices of the liver and spleen to normal levels (Figure 5I,J).

Furthermore, histopathological examination revealed that the model group exhibited high-density tumor cell clusters with an orderly arrangement and darker nuclear staining. In contrast, tumor tissues from the quadrigemine I dose groups and the CDDP group showed a sparse growth pattern with areas of increased apoptosis, characterized by nuclear condensation and fragmentation (Figure 5K, red circle). These results indicated that quadrigemine I reduced the tumor burden in mice.

**Figure 6:**
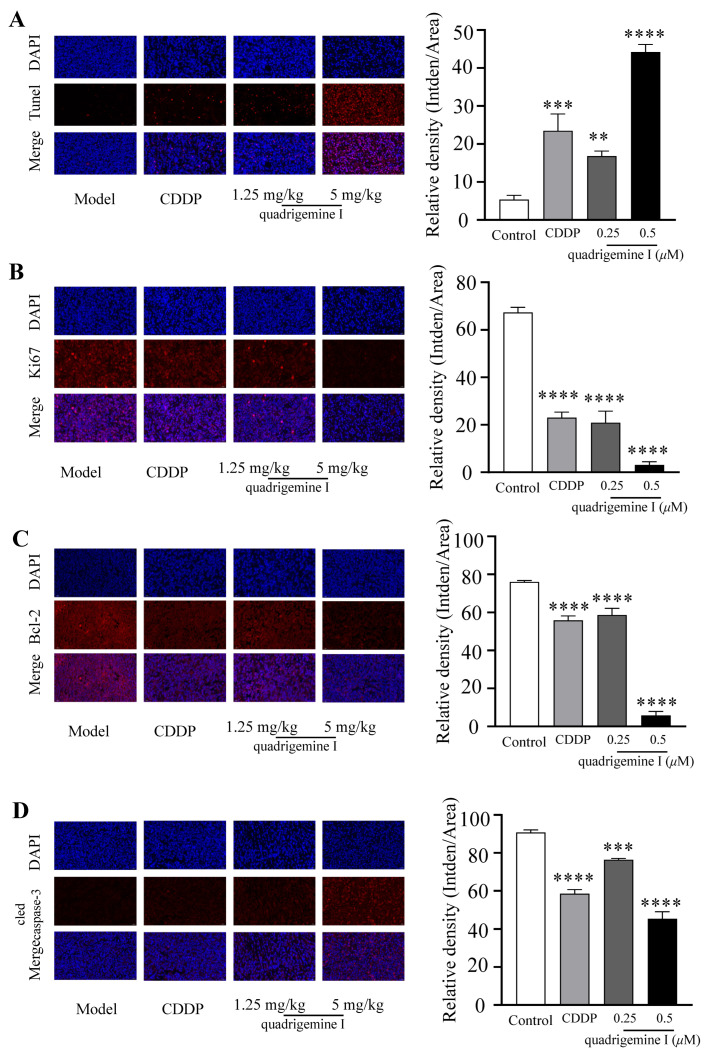
Effects of quadrigemine I on apoptosis in tumor xenograft mice. (**A**) The tumor apoptosis is examined by TUNEL staining, the blue fluorescence (DAPI) in the tumor represents tumor cells, red fluorescence (TUNEL, TMR red) represents apoptotic tumor cells, and the apoptosis index is calculated; the scale bar stands for 10 μm. (**B**) Representative images of immunofluorescence staining of Ki-67 on tumor tissues are shown, and the percentage of Ki-67-positive cells is calculated; the scale bar stands for 10 μm. (**C**) Representative images of immunofluorescence staining of Bcl-2 on tumor tissues are shown, and the percentage of Bcl-2-positive cells is calculated; the scale bar stands for 10 μm. (**D**) Representative images of immunofluorescence staining of caspase-3 on tumor tissues are shown, the blue fluorescence (DAPI) in tumor represents tumor cells, the red fluorescence (TUNEL, TMR red) represents apoptotic tumor cells, and the apoptosis index is calculated; the scale bar stands for 10 μm. The data are shown as the mean ± SD. ** *p* < 0.01, *** *p* < 0.001 and **** *p* < 0.0001 compared with the model group.

Subsequently, to further investigate the mechanism, we performed TUNEL staining and analyzed the expression of apoptosis-related proteins in tumor tissues. We examined the expression of Ki-67, Bcl-2, and cleaved caspase-3 by immunofluorescence staining. As shown in Figure 6A, TUNEL staining of tumor tissues revealed a greater number of apoptotic cells in tumors treated with quadrigemine I compared to those from the model group. Quadrigemine I decreased Ki-67 and Bcl-2 expression while increasing cleaved caspase-3 expression in a dose-dependent manner (Figure 6B–D). These data suggested that quadrigemine I could inhibit tumor growth in tumor xenograft mice by regulating apoptosis pathways in vivo.

**Figure 7:**
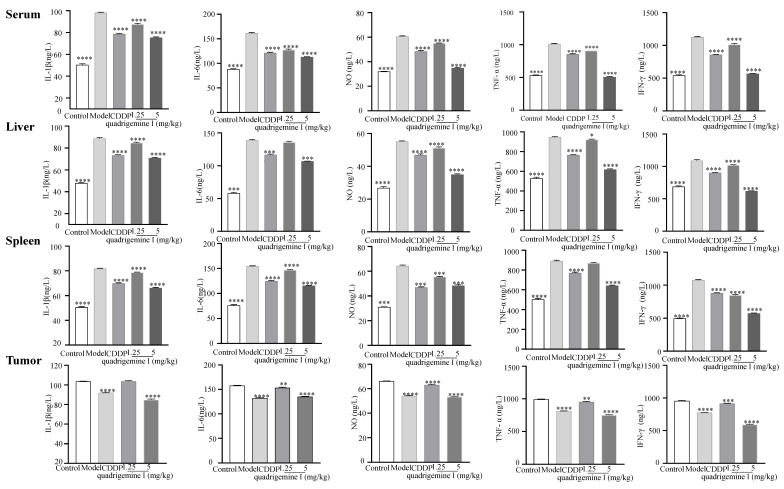
Effect of quadrigemine I on the production of IL-1β, IL-6, NO, TNF-α, and IFN-γ in serum, liver, spleen, and tumor tissue. * *p* < 0.05, ** *p* < 0.01, *** *p* < 0.001, and **** *p* < 0.0001 compared with the model control.

Additionally, we assessed the expression of inflammatory factors in tumor tissues (Figure 7). Compared to the control group, the levels of IL-1β showed a significant increase in the model group: from 50.48 ± 0.84 ng/L to 98.17 ± 0.54 ng/L in serum, from 48.00 ± 0.51 ng/L to 88.65 ± 0.83 ng/L in liver tissue, and from 53.07 ± 0.42 ng/L to 98.13 ± 0.53 ng/L in spleen tissue. However, treatment with quadrigemine I at 1.25 mg/kg and 5 mg/kg effectively inhibited the excessive secretion of inflammatory factors. Specifically, the inhibition rates for IL-1β were 10.79 ± 0.45% and 23.25 ± 0.29% in serum, 4.50 ± 1.52% and 19.92 ± 1.03% in liver tissue, 7.20 ± 0.18% and 18.26 ± 1.09% in spleen tissue, and 0.64 ± 0.60% and 18.43 ± 1.02% in tumor tissue.

Similarly, compared to the control group, the levels of IL-6 in the model group also significantly increased to 161.33 ± 1.49 ng/L, 135.84 ± 1.61 ng/L, 154.66 ± 0.70 ng/L, and 157.68 ± 0.73 ng/L, respectively. After treatment with quadrigemine I (1.25 and 5 mg/kg), the inhibition rates for IL-6 were 20.95 ± 0.30% and 30.00 ± 0.86% in serum, 2.13 ± 1.99% and 22.91 ± 0.71% in liver tissue, 5.10 ± 0.62% and 25.33 ± 0.98% in spleen tissue, and 2.74 ± 0.40% and 14.17 ± 0.09% in tumor tissue.

The levels of NO also showed a significant decrease after treatment with quadrigemine I, with inhibition rates of 9.07 ± 0.99% and 42.24 ± 1.02% in serum, 7.25 ± 1.21% and 36.53 ± 0.82% in liver tissue, 13.77 ± 1.21% and 24.84 ± 1.36% in spleen tissue, and 4.58 ± 1.35% and 19.32 ± 0.47% in tumor tissue.

The levels of TNF-α in the model group were initially 1013.51 ± 7.49 ng/L, 945.65 ± 5.99 ng/L, 890.07 ± 8.30 ng/L, and 992.28 ± 4.90 ng/L, respectively, but significantly decreased after treatment with quadrigemine I, with inhibition rates of 10.28 ± 0.64% and 49.83 ± 0.39% in serum, 2.49 ± 1.11% and 34.59 ± 1.11% in liver tissue, 2.22 ± 0.30% and 27.90 ± 0.09% in spleen tissue, and 3.79 ± 0.87% and 24.48 ± 1.05% in tumor tissue.

Overall, the levels of inflammatory factors in the model group were markedly elevated, while treatment with quadrigemine I resulted in a general reduction in these inflammatory factors across all tissues. The levels of IFN-γ in the model group significantly increased to 1126.89 ± 8.68 ng/L, 1088.81 ± 17.42 ng/L, 1075.39 ± 9.77 ng/L, and 773.86 ± 2.63 ng/L, respectively. After treatment with quadrigemine I, the inhibition rates for IFN-γ were 9.95 ± 1.01% and 49.42 ± 0.71% in serum, 6.35 ± 2.05% and 43.13 ± 0.60% in liver tissue, 21.21 ± 0.21% and 46.59 ± 0.66% in spleen tissue, and 3.97 ± 0.88% and 38.03 ± 0.22% in tumor tissue. These results indicated that quadrigemine I can alleviate tumor-induced inflammatory responses in vivo.

## 3. Discussion

Quadrigemine I is a natural indole alkaloid derived from *P*. *pilifera*. To our knowledge, this research represents, for the first time, the inhibitory effect of quadrigemine I on lymphoma cells. Our study investigated the effects of quadrigemine I on the proliferation, apoptosis, ROS generation, and DNA damage of SR cells. The results indicated that quadrigemine I inhibited proliferation, increased ROS generation, and induced cell apoptosis and DNA damage in SR cells. The CCK-8 assay showed that quadrigemine I inhibited the proliferation of SR cells with an IC_50_ of 0.46 ± 0.12 µM. Our results showed that quadrigemine I exhibited a low IC_50_ against SR cells and displayed no cytotoxicity toward HUVECs (Figure 1). One major limitation of this study is the use of only two lymphoma cell lines—SR cells representing T cell lymphoma and Raji cells representing B cell lymphoma. Although these lines provide initial insight into the effects of quadrigemine I on different lymphocyte-derived tumor types, they do not fully capture the heterogeneity and complexity of lymphomas. The differences observed may be influenced by intrinsic characteristics specific to these cell lines rather than representative of broader lymphoma subtypes. Future studies will aim to validate these findings across a larger panel of lymphoma cell lines, including those of various genetic backgrounds and clinical relevance, as well as primary patient-derived tumor cells.

Using flow cytometry, we found that quadrigemine I treatment dramatically promoted apoptosis in a dose-dependent manner. Notably, the results (Figure 1F) showed minimal effect of quadrigemine I on the proportion of early apoptotic cells, while a significant increase was observed in late apoptotic populations. This suggests that quadrigemine I may rapidly drive cells from a viable state to late apoptosis or secondary necrosis, bypassing the early apoptotic phase. One possible explanation is that the overproduction of ROS and the induction of DNA damage trigger a robust and acute pro-apoptotic response, leading to mitochondrial dysfunction and rapid activation of caspase cascades [11]. Alternatively, the selected time point for apoptosis detection may have primarily captured the late phase of apoptosis [12]. Future studies involving time course experiments will be necessary to further delineate the dynamics of quadrigemine I-induced apoptosis.

Additionally, the mechanism of quadrigemine I-induced cell apoptosis is not fully understood. Excessive cellular levels of ROS cause damage to proteins, nucleic acids, lipids, membranes, and organelles, leading to the activation of cell death processes [13]. DNA strand breaks (DSBs) are the most severe incidence of DNA damage, which can cause cell apoptosis [14]. An effective DNA repair process could restore DNA integrity, whereas unrepaired DNA damage might trigger cell death, or damaged cells might evade apoptosis, potentially leading to cancer [15]. In this study, we observed a significant increase in intracellular ROS levels following quadrigemine I treatment, which correlated with enhanced DNA damage, as indicated by the comet assay (Figure 1). Excessive ROS can cause oxidative modifications to DNA, including strand breaks and base damage, thereby compromising genomic integrity. This DNA damage activates the ATM/ATR-mediated DNA damage response (DDR), which in turn can trigger the intrinsic apoptotic pathway through mitochondrial dysfunction, cytochrome c release, and caspase activation. Our findings suggest that ROS-induced DNA damage is a critical upstream event leading to apoptosis in quadrigemine I-treated SR cells. This mechanistic cascade is consistent with previous reports [16].

Transcriptome analysis showed that quadrigemine I altered the expression of thousands of genes in SR cells (Figure 2). These DEGs were significantly enriched in multiple tumor-associated signaling pathways. These key genes, including MAP3K8, MAPK10, FOS, ELF3, GADD45A, HSPA5, and UCN2, have been shown in previous studies to be closely associated with apoptosis [14,15]. For example, HSPA5 (heat shock protein family A (Hsp70) member 5) can play diverse functional roles in cell viability, proliferation, apoptosis, attachment, and innate and adaptive immunity regulation, which can lead to various diseases, including cancers and coronavirus disease 2019 (COVID-19) [17]. HSPA5 expression was significantly upregulated in 14 types of cancer, including cholangiocarcinoma, colon adenocarcinoma, esophageal carcinoma, diffuse large B cell lymphoma, pancreatic adenocarcinoma, and others. These findings suggest that HSPA5 is a crucial cancer marker, highly expressed in most malignant tumors, and that targeted therapy targeting HSPA5 may be a feasible approach for cancer treatment [18]. Liu et al. found that after treating melanoma cells with CDDP, the expression of GADD45A was upregulated in a dose- and time-dependent manner. The inhibition of GADD45A made melanoma cells more sensitive to cisplatin, enhanced cisplatin-induced DNA damage, and induced apoptosis [19]. Studies have also shown that miR-335-5p can exert its suppressive effect on gastric cancer by targeting MAPK10. The underlying mechanism is that the downregulation of MAPK10 inhibits the proliferation, migration, and invasion of gastric cancer cells and induces apoptosis [20]. Studies have found that ELF3 is differentially expressed in non-small cell lung cancer. In lung cancer cells harboring K-ras mutations and EGFR L858R/T790M mutations, the knockdown of ELF3 significantly increases cell apoptosis [21]. Ucn2 is a specific ligand for the corticotropin-releasing factor (CRF) receptor CRFR2. Previous studies have shown that Ucn2 could suppress tumor growth predominantly by the inhibition of tumor vascularization, but also potentially through direct effects on tumor cell proliferation [22]. To the best of our knowledge, we report, for the first time, that quadrigemine I upregulated the expression of UCN2 and downregulated the expression of MAP3K8, MAPK10, FOS, ELF3, GADD45A, and HSPA5 on SR cells. These findings provide novel insights into the molecular mechanisms underlying the potential role of quadrigemine I in cellular stress response, survival, and apoptosis regulation (Figure 3 and Figure 4). For these key and novel targets, we will further use RNA interference (RNAi) and overexpression techniques to validate the importance of these targets in SR cell apoptosis. Notably, transcriptomic enrichment analysis revealed that genes in the ERBB signaling pathway were significantly modulated in response to quadrigemine I treatment, particularly in SR cells. Although the ERBB pathway is not typically regarded as a major proliferative signal in normal lymphocytes, its altered activation in lymphoma cells may represent a disease-specific adaptation or a drug-induced response. Additionally, we will conduct in vivo studies to further investigate the role and mechanism of quadrigemine I in the treatment of lymphoma.

Using NOD/SCID xenograft mice as a model, it was demonstrated that quadrigemine I treatment significantly inhibited tumor growth without exhibiting toxicity (Figure 5). Furthermore, histopathological examination, immunofluorescence, and TUNEL staining analysis revealed that quadrigemine I treatment caused tumor cell apoptosis (Figure 6). However, the specific molecular targets of quadrigemine I and its detailed mechanism in the apoptosis signaling pathway require further investigation. Future research could explore the interaction of quadrigemine I with other apoptosis-regulating proteins (such as Bax, p53, etc.), as well as its long-term efficacy and safety in vivo and vitro [23]. In this study, we evaluated the anti-tumor efficacy of quadrigemine I in vivo using two dosage groups. The selection of these doses was based on preliminary in vivo safety evaluations and the limited availability of the compound at the time of extraction. While both doses demonstrated significant tumor-suppressive effects, we acknowledge that the current study does not fully address the dose–response relationship of quadrigemine I in vivo. Future studies will involve additional dose groups and expanded pharmacodynamic assessments to comprehensively determine the optimal dosing strategy for quadrigemine I in lymphoma.

Inflammation is closely related to the development of cancer, and inhibiting chronic inflammation or accelerating its resolution can improve the overall survival rate in cancer therapy [24]. The mechanisms by which inflammation promotes cancer include inflammatory cytokines activating intracellular signaling pathways to promote tumor cell proliferation and inhibit apoptosis; inflammatory mediators inducing the expression of angiogenic factors to promote tumor angiogenesis and facilitate tumor metastasis; and inflammatory processes in the tumor microenvironment causing immune cell dysfunction, the accumulation of immunosuppressive cells, and the inhibition of cytotoxic T cell activity, thereby assisting tumor cells in immune escape [25]. A decrease in key inflammatory biomarker (such as IL-6, TNF-α, IFN-γ, etc.) levels can reshape the tumor immune microenvironment. For example, Zhao et al. found that cancer-associated adipocytes (CAAs) secrete large amounts of IL-6, which activates STAT3 to induce macrophage polarization toward the M2 type, promoting breast cancer progression. This indicates that IL-6 plays an important role in inducing M2 polarization of tumor-associated macrophages (TAMs) [26]. Other studies have shown that IL-1β can promote MDSC accumulation, thereby driving tumor progression. Therefore, reducing inflammation may decrease the number of Myeloid-Derived Suppressor Cells (MDSCs) and slow down tumor progression [27]. TNF-α upregulation is involved in the angiogenesis process in the tumor microenvironment, affecting the tumor’s nutrient supply and waste removal, creating favorable conditions for tumor growth, and promoting tumor cell migration and invasion [28]. Nitric oxide (NO) is involved in the process by which tumor cells acquire stem-cell-like abilities, helping them escape immune system recognition. NO can suppress the activity of cytotoxic T cells, natural killer cells, and other immune cells, reducing their ability to recognize and kill tumor cells. Therefore, downregulating NO expression may help inhibit tumor growth [29]. IFN-γ in the tumor microenvironment induces the production of immunosuppressive cells, causes immune cell functional exhaustion, and can also induce tumor cell metabolic reprogramming, enabling them to adapt to immune pressure, thereby promoting tumor cell immune escape [30]. These findings highlight the therapeutic potential of quadrigemine I in regulating both tumor growth and the associated inflammatory response. Quadrigemine I was observed to modulate the inflammatory microenvironment, a hallmark of tumor progression. Our data indicated that treatment with quadrigemine I significantly reduced the secretion of key pro-inflammatory cytokines, such as TNF-α, IL-6, and IL-1β, in various tissues, including serum, liver, spleen, and tumor tissues (Figure 7). This anti-inflammatory effect is consistent with the mechanisms proposed for other natural compounds that target inflammatory pathways in cancer [31].

Although our study demonstrates the therapeutic efficacy of quadrigemine I both in vitro and in vivo, we acknowledge that the lack of pharmacokinetic and biodistribution data remains a limitation. Given that quadrigemine I was administered systemically, it is essential to investigate its absorption, distribution, metabolism, and excretion (ADME) characteristics in future studies. These parameters will provide critical insights into its bioavailability, tissue targeting, and safety profile, all of which are crucial for optimizing dosing strategies and advancing quadrigemine I toward clinical application. Ongoing and future work in our laboratory will include detailed pharmacokinetic analyses and imaging-based biodistribution studies to address this gap and support the translational potential of quadrigemine I.

## 4. Materials and Methods

### 4.1. Chemicals and Reagents

^1^D and ^2^D NMR spectra were performed on a Bruker DRX–500 spectrometer (Bruker BioSpin GmBH, Rheinstetten, Germany). The fractions were analyzed using thin-layer chromatography (TLC, GF254, Qingdao Marine Chemical Ltd., Qingdao, China), and the spots were detected using Dragendorff’s reagent and 10% H_2_SO_4_ in ethanol. 2′,7′-Dichlorofluorescin diacetate (DCFH-DA) was purchased from Sigma-Aldrich (Shanghai, China). The Cell Counting Kit-8 (CCK-8) was purchased from UElandy Biotech Co., Ltd. (Suzhou, China). The Annexin V fluorescein isothiocyanate (FITC)/propidium iodide (PI) apoptosis detection kit was purchased from 4A Biotech Co., Ltd. (Beijing, China). Phosphate-buffered saline (PBS) was obtained from Gibco (Grand Island, NY, USA).

### 4.2. Plant Material

The leaves of *P. pilifera* were collected in July 2022 from Mengna, Yunnan Province, China. The plant material with species number Cheng2022072001 was identified by Prof. Yaping Liu from the Kunming University of Science and Technology.

### 4.3. Extraction, Isolation, and Structure Characterization of Quadrigemine I

The air-dried leaves of *P. pilifera* (5.4 kg) were extracted with 90% ethanol aqueous solution three times. After filtration by nylon gauze, the extraction solutions were collected and evaporated by a rotary evaporator to yield an extract. Then, the extract was applied to a silica gel column, eluted by a CHCl_3_-MeOH-NH_3_·H_2_O solution system (10:1:0.3), and further purified by an MPLC column with RP–C18 chromatography (MeOH/H_2_O, 50:50) to obtain quadrigemine I (63 mg). Finally, quadrigemine I (10 mg) was dissolved in a DMSO solution for NMR analysis. The ^1^H and ^13^C NMR spectra were recorded on a Bruker 500 MHz spectrometer. The chemical shifts (δ) were expressed in parts per million and coupling constants (J) in Hz.

^1^H NMR (500 MHz, DMSO-D6) δ 1.82-3.31 (16H, m), 2.28, 2.33, 2.37, 2.49 (12 H, 4×N-CH3), 4.89 (1H, s), 4.84 (1H, s), 4.78 (1H, s), 4.58 (1H, s), 5.58 (1H, s), 5.69, (1H, s), 7.01 (1H, s), 7.08, (1H, s), 7.15, (4H, s), and 7.22 (1H, s). ^13^C NMR (125 MHz, DMSO-D6) δ: 152.1, 151.5, 149.8, 148.6, 132.9, 132.1, 131.6, 127.8, 127.7, 125.7, 125.6, 124.8, 124.4, 123.5, 123.2, 117.2, 116.6, 116.4, 115.9, 107.0, 106.9, 86.4, 85.3, 83.1, 82.2, 62.9, 62.7, 59.8, 59.3, 51.9, 37.9, 37.6, 36.9, and 35.1. By comparison with the reported data, this compound was characterized as quadrigemine I (Figure 1A) [32].

### 4.4. Cell Culture

SR cells were purchased from the American Type Culture Collection (ATCC). Human umbilical vein endothelial cells (HUVECs) were acquired from Wuhan Servicebio technology Co., Ltd. (Wuhan, China), and Raji cells were obtained from Wuhan Servicebio technology Co., Ltd. (Wuhan, China). In this study, two lymphoma cell lines were used: SR cells, derived from T cell lymphoma, and Raji cells, derived from B cell lymphoma. These cell lines were selected to represent different lymphoma subtypes for investigating the effects of quadrigemine I on lymphocyte malignancy.

SR cells were cultured in an RPMI-1640 medium supplemented with 10% fetal bovine serum (FBS) and 1% penicillin/streptomycin (Gibco, Grand Island, NY, USA). HUVECs were cultured in a ScienCell ECM 1001 endothelial cell medium supplemented with 5% FBS, 1% endothelial cell growth supplement (ECGS), and 1% penicillin/streptomycin. Media and supplements were purchased from ScienCell research laboratories (Carlsbad, CA, USA). All cells were maintained at 37 °C with 5% CO_2_.

### 4.5. Cell Viability Assay

The CCK-8 assay was used to measure cytotoxicity. Briefly, HUVECs (5 × 10^3^ cells/well) were seeded in 96-well plates for 24 h and then treated with quadrigemine I for 20 h. SR cells were cultured in 96-well plates (5 × 10^4^ cells/well) and treated with quadrigemine I or cisplatin (CDDP) for 44 h. Then, 10 µL of CCK-8 solution was added to each well and incubated at 37 °C for 4 h. Absorbance was measured at 450 nm using a microplate reader (SpectraMax M5, Molecular Devices, San Francisco, CA, USA).

### 4.6. Determination of Intracellular Reactive Oxygen Species (ROS)

SR cells (1 × 10^6^ cells/well) were seeded into 6-well plates and treated with quadrigemine I (0, 0.125, 0.25, 0.5 µM) or CDDP (8 µM) for 48 h. After treatment, the cells were harvested, washed twice with cold PBS, and incubated with 10 µM DCFH-DA in the dark for 30 min. Following incubation, the cells were rinsed twice and analyzed by flow cytometry (Guava^®^ easyCyte 6-2L, Millipore, Billerica, MA, USA).

### 4.7. Comet Assay

SR cells and HUVECs were treated with quadrigemine I in 6-well plates for 48 h and then harvested. Single-cell suspensions were prepared and collected for the assay. After gel preparation, the cells were lysed in a comet assay lysis buffer at 4 °C for 1 h. Single-cell electrophoresis was performed at 25 V and 300 mA for 20 min. Finally, the cells were stained with DAPI, and data analysis was conducted using CASP software (version 1.2.3 beta 1).

### 4.8. Cell Apoptosis Assays

SR cells (1 × 10^6^ cells/well) were seeded into 6-well plates and cultured for 48 h. After the incubation period, the cells were harvested, rinsed three times with cold PBS, and then resuspended in 100 µL of binding buffer. Next, 5 µL of Annexin V-FITC was introduced, and the cells were incubated in the dark for 5 min. Thereafter, 10 µL of propidium iodide (PI, 20 µg/mL) and 200 µL of a binding buffer were added to the cell suspension. Apoptosis levels were determined by flow cytometry (Guava^®^ easyCyte 6-2L, Millipore, Billerica, MA, USA).

### 4.9. RNA-Seq Analysis

SR cells (1 × 10^6^ cells/well) in the logarithmic growth phase were treated with quadrigemine I (0.125, 0.25, 0.5 µM) or CDDP (8 µM) in 6-well plates for 48 h. The cells were then collected, washed once with PBS, and lysed using TRIzol reagent (Thermo Fisher Scientific, Waltham, MA, USA). The obtained cDNA libraries were sequenced by Beijing Yijian Testing Technology Co., Ltd. (Beijing, China).

### 4.10. Real-Time Quantitative PCR (RT-qPCR)

Total RNA was extracted using TRIzol reagent (Thermo Fisher Scientific, Waltham, MA USA). cDNA synthesis was conducted using the cDNA synthesis kit (TIANGEN, Beijing, China) in a 20 μL reaction volume. Quantitative PCR (qPCR) was carried out with SYBR Green Master Mix (TIANGEN, Beijing, China), and the reactions were performed and analyzed on a CFX Connect Real-Time System (Bio-Rad, Hercules, CA, USA). The relative expression levels of target genes were determined using the ΔΔCt method [33]. The qPCR primers (Table 1) were designed and synthesized by Sangon Biotech (Shanghai, China).

### 4.11. Safety Evaluation in Mice

Twelve ICR mice were randomly divided into two groups (5 mg/kg dose group and control group), with six mice in each group. Each mouse was intraperitoneally injected with 0.2 mL of the solution daily. During the experiment, the reactions of each animal were observed before and after administration. Body weight changes were measured every two days. At the end of the experimental period, organ indices, histopathological changes, and hematological parameters were evaluated in the mice.

### 4.12. In Vivo Anti-Tumor Evaluation

Female NOD/SCID mice aged 4 weeks were purchased from Sybef Biotechnology Co., Ltd. (Beijing, China). The mice were kept under specific pathogen-free (SPF) conditions at Yunnan University with a 12 h light/dark cycle. They were given sufficient food and water, and all experimental procedures followed the guidelines approved by the Yunnan University Laboratory Animal Care Committee. The Ethics Committee of Yunnan University approved the animal use and experimental protocols (permit number YNU20251207).

For xenograft tumor studies, SR cells (1 × 10^7^) were subcutaneously injected into NOD/SCID mice at a volume of 100 µL. Tumor volume was calculated using the formula length × (width^2^)/2. When the tumor volume reached 100 mm^3^, the mice were randomly divided into groups (n = 6) and treated with intraperitoneal injections of normal saline (NS), CDDP (5 mg/kg), or quadrigemine I (1.25 and 5 mg/kg) for 14 days.

### 4.13. Hematoxylin and Eosin (H&E) Staining, Immunofluorescence, and Terminal Deoxynucleotidyl Transferase dUTP Nick End Labeling (TUNEL) Assays

Tumor tissues were removed from the xenograft mice and fixed in 4% paraformaldehyde for 24 h at room temperature, followed by dehydration and paraffin embedding. The tumor tissues were sectioned into 5 μM thick slices and stained with hematoxylin and eosin solution. The stained sections were scanned using a pathology slide scanner (Nikon, Tokyo,Japan), and the images were viewed using CaseViewer 2.4 software.

For immunofluorescence analysis, the sections were incubated overnight with primary antibodies, including cleaved cysteine-aspartic acid protease 3 (cleaved caspase-3, Affinity, San Francisco, CA, USA), B cell lymphoma 2 (Bcl-2, Servicebio, Wuhan, China), and Kiel 67 (Ki-67, Servicebio, Wuhan, China). After washing, the sections were incubated with fluorescent Cy3 goat anti-rabbit IgG secondary antibodies (Servicebio, Wuhan, China). Nuclei were counterstained with DAPI, and fluorescent signals were observed under a fluorescence microscope (Nikon, Tokyo, Japan). The images were analyzed using ImageJ software (version 1.8.0).

The TUNEL assay was performed according to the protocol provided with the TUNEL apoptosis detection kit (Servicebio, Wuhan, China). Briefly, paraffin-embedded tumor sections were deparaffinized and incubated with proteinase K to remove nuclear proteins. The sections were then incubated with TdT and dUTP enzyme reaction mixtures. Nuclei were stained with DAPI, and the sections were observed under a fluorescence microscope (Nikon, Tokyo, Japan). The percentage of TUNEL-positive cells was quantified using ImageJ software (version 1.8.0).

### 4.14. ELISA Analysis of Interleukin (IL)-1β, IL-6, Nitric Oxide (NO), Tumor Necrosis Factor (TNF)-α, and Interferon (IFN)-γ

The mice were euthanized, and the tumor, liver, and spleen tissues, along with serum samples, were harvested. Following this, the concentrations of IL-1β, IL-6, NO, TNF-α, and IFN-γ in the samples were quantified using ELISA kits, which were procured from Jiangsu Meimian Industrial Co., Ltd. (Yancheng, China).

### 4.15. Statistical Analysis

Data are presented as mean ± standard deviation (SD) from three independent experiments. Statistical analysis was performed using one-way ANOVA followed by Dunnett’s multiple comparisons test. Significance levels were defined as * *p* < 0.05, ** *p* < 0.01, *** *p* < 0.001, and **** *p* < 0.0001.

## 5. Conclusions

In conclusion, our study suggests that quadrigemine I is effective in inhibiting lymphoma cell proliferation while remaining safe for normal human cells. At a dose of 5 mg/kg, quadrigemine I significantly suppressed tumor growth in xenograft NOD/SCID mice. Its mechanism of action may be associated with immune evasion, inflammation, and apoptosis pathways. These findings highlight quadrigemine I as a promising anti-proliferative agent for the treatment of lymphomas.

## Figures and Tables

**Table 1:** The primers used for RT-qPCR analysis.

Gene	Species	Primer Sequences
*MAP3K8*	human	F:5′ AGCAACATTGTTTTCATGTCCAC3′
		R:5′ AGCAACATTGTTTTCATGTCCAC3′
*MAPK10*	human	F:5′ AGCAACATTGTTTTCATGTCCAC3′
		R:5′ AGCAACATTGTTTTCATGTCCAC3′
*FOS*	human	F:5′CTTCCTGGAGCAGTGTGGAG3′
		R:5′ AGCAACATTGTTTTCATGTCCAC3′
*UCN2*	human	F:5′ AGAAGCAGCTGGTGGCG3′
		R:5′ CACTGGGACAACCAGGACTC3′
*ELF3*	human	F:5′ AAGGCGTCTTCAAGTTCC3′
		R:5′ TAGGTCATGTTGCTGTTCTT3′
*GADD45A*	human	F:5′ GACGAATCCACATTCATCTC3′
		R:5′ GACGAATCCACATTCATCTC3′
*HSPA5*	human	F:5′ GTTACAATCAAGGTCTAT3′
		R:5′ CATTCACATCTATCTCAA3′
*GAPDH*	human	F:5′ AGTGGCAAAGTGGAGATT3′
		R:5′ GTGGAGTCATACTGGAACA3′

## Data Availability

The original contributions presented in this study are included in the article/Supplementary Materials. Further inquiries can be directed to the corresponding author.

## Supplementary Materials

The following supporting information can be downloaded at https://www.mdpi.com/article/10.3390/ijms26104848/s1.

## Abbreviations

The following abbreviations are used in this manuscript:

DCFH-DA	2′,7′-Dichlorofluorescin diacetate
2ATF2	Activating transcription factor
AKT3	AKT serine/threonine kinase 3
ATCC	American Type Culture Collection
BCL2A1	BCL2-related protein A1
Raji	Burkitt lymphoma cells
CCK-8	Cell Counting Kit-8
CDDP	Cisplatin
CRF	Corticotropin-releasing factor
DEGs	Differentially expressed genes
DSBs	DNA strand breaks
ELF3	E74-like ETS transcription factor 3
ERBB3	Erb-B2 receptor tyrosine kinase 3
FBS	Fetal bovine serum
FITC	Fluorescein isothiocyanate
GADD45A	Growth arrest and DNA damage inducible alpha
HSPA5	Heat shock protein family A (Hsp70) member 5
H&E	Hematoxylin and eosin
HL-60	Human acute promyelocytic leukemia cells
SR	Human large-cell immunoblastic lymphoma cells
HUVECs	Human umbilical vein endothelial cells
IFN	Interferon
IL	Interleukin
MAPK	Mitogen-activated protein kinase
MAP3K8	Mitogen-activated protein kinase 8
NO	Nitric oxide
PIK3CD	Phosphatidylinositol-4,5-bisphosphate 3-kinase catalytic subunit delta
PI	Propidium iodide
ROS	Reactive oxygen species
RT-qPCR	Real-time quantitative PCR
RNA-seq	RNA sequencing
SPF	Specific pathogen free
TCR	T cell receptor
TUNEL	Terminal deoxynucleotidyl transferase dUTP nick end labeling
TGFA	Transforming growth factor alpha
TNF	Tumor necrosis factor
UCN2	Urocortin 2
