# Supplementary material for: Therapeutic Potential of Quadrigemine I Against Lymphoma: Mechanistic Insights from Cell Lines and Xenograft Models Demonstrating DNA Damage, Oxidative Stress, and Pathway Regulation
Source: Int J Mol Sci. 2025 May 19;26(10):4848. doi: 10.3390/ijms26104848 (PMC12112571; doi:10.3390/ijms26104848)
Supplement: Supplementary file 1 [file ijms-26-04848-s001.zip › ijms-3620589-supplementary.pdf]

Table S1. Hematological analysis results of mice intraperitoneal injection with quadrigemine I for 14 days.

Haematological index	Control	quadrigemine I (5mg/kg)
WBC (10^9/L)	4.70 ± 0.44	5.73 ± 0.32
RBC (10^{12}/L)	8.22 ± 0.08	8.21 ± 0.09
HGB (g/L)	118.33 ± 12.10	119.67 ± 56.09
MCV (f L)	42.67 ± 0.71	41.17 ± 0.42
PLT (f L)	975.00 ± 58.66	1056.00 ± 32.60
Lym (10^9/L)	2.63 ± 0.23	3.43 ± 0.15
GR (10^9/L)	1.33 ± 0.15	1.30 ± 0.17
Lym (%)	56.37 ± 0.47	50.67 ± 0.40
Mid (%)	15.33 ± 0.40	14.80 ± 0.26
GR (%)	28.30 ± 0.60	34.53 ± 0.15
HCT (%)	39.90 ± 0.35	39.87 ± 0.38
MCHC (g/L)	330.33 ± 4.50	330.67 ± 4.28
RDW_CV (%)	15.60 ± 0.35	15.70 ± 0.30
MPV (f L)	4.57 ± 0.15	4.30 ± 0.10
PCT (%)	0.74 ± 0.06	0.77 ± 0.01
PDW (f L)	23.67 ± 0.65	23.10 ± 0.56

Table S2. Analysis of biochemical indexes in mice after intraperitoneal injection of quadrigemine I for 14 days.

Blood biochemical indices	Control	quadrigemine I (5mg/kg)
ALT (U/L)	42.77 ± 6.96	42.16 ± 5.39
AST (U/L)	120.06 ± 2.11	121 ± 1.17
ALB (g/L)	27.67 ± 1.39	27.04 ± 2.01
TP (g/L)	47.59 ± 4.05	47.72 ± 5.46
ALP (U/L)	104.98 ± 7.96	105.99 ± 7.56
TC (mmol/L)	1.91 ± 0.46	1.88 ± 0.35
TG (mmol/L)	1.07 ± 0.06	1.06 ± 0.07
BUN (mmol/L)	32.79 ± 0.39	32.93 ± 0.53
CR (μmol/L)	15.10 ± 1.47	14.60 ± 1.46
K (mmol/L)	5.84 ± 0.52	5.84 ± 0.46
Na (mmol/L)	667.59 ± 10.12	663.50 ± 7.05
CL (mmol/L)	649.44 ± 13.01	644.37 ± 7.89

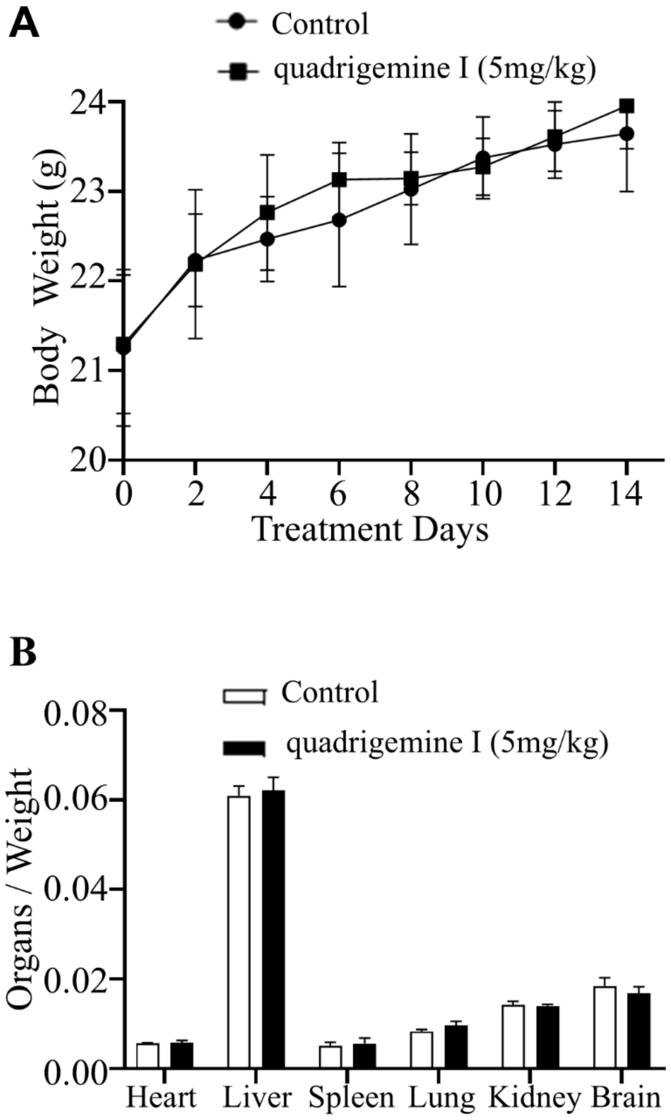

Figure S1

(A) Body weight following continuous intraperitoneal administration of 5 mg/kg to the mice over a 14-day period. (B) Ratio of organs to body weight following continuous intraperitoneal administration of 5 mg/kg to the mice over a 14-day period.

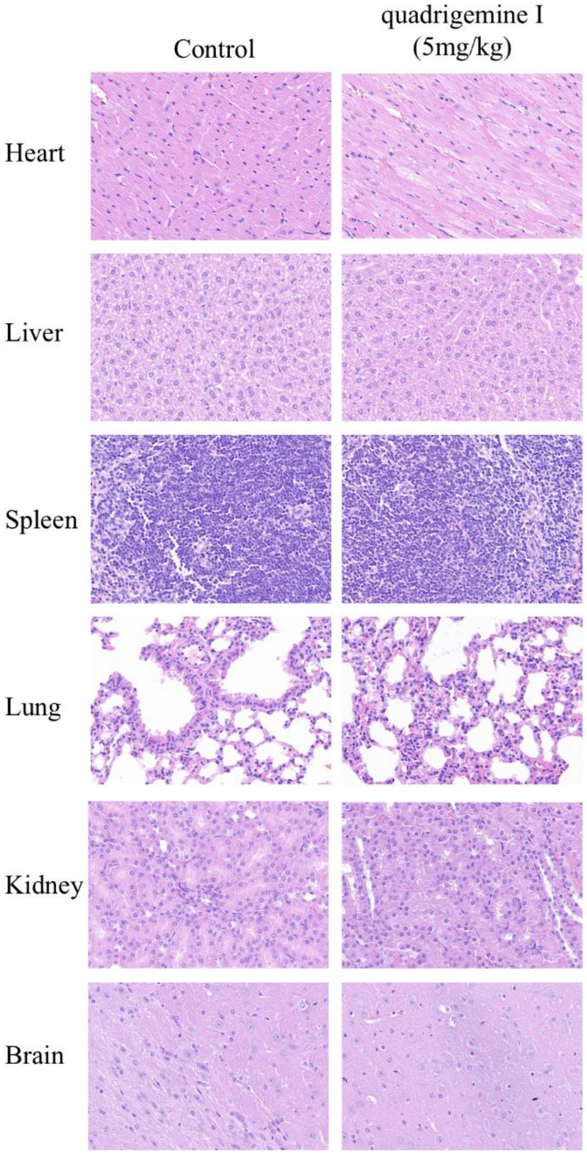

Figure S2

H&E staining of Heart, liver, Spleen, Lung, Kidney and Brain.
